# Paradoxical massive pulmonary thromboembolism in a postpartum woman with factor VII deficiency with bleeding tendency: A case report

**DOI:** 10.1097/MD.0000000000033437

**Published:** 2022-04-07

**Authors:** Donghoon Kang, Hojeong Cha, Sung Eun Park, Jong-Hwa Ahn, Ji Kwon Park, Iyun Kwon, Ji Eun Park

**Affiliations:** a Department of Thoracic and Cardiovascular Surgery, Gyeongsang National University School of Medicine and Gyeongsang National University Changwon Hospital, Changwon, Korea; b Department of Radiology, Gyeongsang National University School of Medicine and Gyeongsang National University Changwon Hospital, Changwon, Korea; c Department of Internal Medicine, Gyeongsang National University School of Medicine and Gyeongsang National University Changwon Hospital, Changwon, Korea; d Department of Obstetrics and Gynecology, Hanyang University Hanmaeum Changwon Hospital, Changwon, Korea; e Department of Obstetrics and Gynecology, Gyeongsang National University School of Medicine and Gyeongsang National University Changwon Hospital, Changwon, Korea; f Institute of Health Science, Gyeongsang National University, Jinju, Korea

**Keywords:** cesarean delivery, Factor VII (FVII) deficiency, premature rupture of membranes, pulmonary thromboembolism

## Abstract

Factor VII (FVII) deficiency is an inherited bleeding disorder, and women with FVII deficiency are at risk of gynecological bleeding and postpartum hemorrhage. There have been no reports of pulmonary embolism in a postpartum woman with FVII deficiency as of yet. We report a case of postpartum massive pulmonary embolism with FVII deficiency.

**Patient concerns:**

A 32-year-old woman visited the hospital with premature rupture of membranes at 24 weeks and 4 days of gestation. She was diagnosed with FVII deficiency in an additional blood test after her laboratory results at admission included an increased prothrombin time and international normalized ratio abnormalities. After 12 days of pregnancy maintenance treatment, an emergency cesarean delivery was performed due to uncontrolled preterm labor. The day after the operation, she suffered a sudden loss of consciousness and cardiac arrest, and after she received 1 cycle of cardiopulmonary resuscitation, she was moved to the intensive care unit.

**Diagnoses:**

She was diagnosed with massive pulmonary thromboembolism with heart failure by chest enhanced computed tomography, C-echo, and angiography.

**Interventions:**

She was successfully treated with the early application of extracorporeal membrane oxygenation, catheter-guided thrombectomy, and anticoagulants.

**Outcomes:**

There were no major sequelae over 2 months of follow-up.

**Lessons:**

FVII deficiency does not protect against thrombosis. Due to the high thrombotic risk after childbirth, the risk of thrombosis should be recognized, and thromboprophylaxis should be considered if additional obstetric thrombotic risk factors are present.

## 1. Introduction

Factor VII (FVII) deficiency is the most common of the rare inherited coagulation disorders, with an estimated prevalence of 1 in 500,000.^[1]^ With an autosomal recessive inheritance pattern, the clinical phenotype ranges from asymptomatic to a high-risk of severe hemorrhage with life-threatening and disabling symptoms.^[2]^

Surprisingly, there are some rare case reports of thrombosis in FVII deficiency. Among the factors related to these episodes, FVII replacement accounts for the majority, other factors include surgery or trauma, and there are reports of spontaneous thrombosis without obvious precipitating factors.^[3,4]^

About 1 in 1000 to 3000 pregnancies are affected by pulmonary embolism (PE).^[5]^ The most severe form, high-risk or massive PE, causes hemodynamic instability with acute extensive pulmonary artery obstruction, and the mortality rate in these hemodynamically unstable patients is 52%.^[6]^ High-risk pregnancy-related PE is responsible for approximately 10% to 15% of all maternal deaths and is a leading cause of maternal death in the United States and the United Kingdom.^[7–9]^

Herein, we report a case of massive PE that occurred the day after cesarean delivery in a woman with FVII deficiency with bleeding tendency who was treated conservatively for preterm premature rupture of membrane (PPROM) and cesarean section. This is the first report on pregnancy-related PE associated with inherited FVII deficiency. The patient was successfully treated with early extracorporeal membrane oxygenation (ECMO), catheter-guided thrombectomy, and anticoagulants.

## 2. Case report

A healthy 32-year-old primigravida was admitted to the hospital due to premature rupture of membranes at 24 weeks and 4 days of gestation. There was no history of hypertension during pregnancy, diabetes, or a family history of cardiomyopathy or sudden death. Her body mass index was 30.74 (92 kg and 1.73 m). Prolonged prothrombin time (17.2 seconds [11.9–14.3 seconds]) and international normalized ratio (1.38 [0.8–1.2]) values were observed in blood coagulation tests performed at the time of hospital admission, and other blood tests, including activated partial thromboplastin time (30.8 seconds [29.1–43.5 seconds]) were within the normal range. Her medical history included an operation for an accessory navicular bone 10 years before this presentation; she heard that there was a problem with blood clotting at that time, but she did not know the exact diagnosis. Therefore, we performed an additional coagulopathy workup and found her serum FVII coagulant level to be 27%, which led to a diagnosis of inherited FVII deficiency. The rest of the coagulopathy test results, including lupus anticoagulant, beta 2 glycoprotein, anticardiolipin, factor V mutation, and prothrombin gene mutation, were negative.

Since the premature rupture of membranes occurred at an extremely early gestational age, she was hospitalized for conservative management. The standard conservative management protocol for PPROM was initiated. This included intravenous (IV) ceftriaxone 2 g once a day, IV metronidazole 500 mg 3 times a day, and oral clarithromycin 3 times a day for 1 week. A course of intramuscular betamethasone sodium phosphate (12 mg × 2) and IV magnesium sulfate for fetal neuroprotection was initiated and administered for 2 days. Owing to the FVII deficiency, low-molecular-weight heparin for thromboprophylaxis was not used, but antithrombotic stockings were applied. Routine blood ordered every 3 days indicated stable levels of hemoglobin, white blood cells, and C-reactive protein.

Seven days after the diagnosis of PPROM, regular uterine contractions began, and changes in cervical length were observed. IV atosiban was administered accordingly. Thirteen days after the diagnosis of PPROM (26 weeks and 2 days of gestation), an emergency cesarean section was performed because of uncontrolled preterm labor and breech fetal position. She delivered a female newborn (weight: 830 g; Apgar scores 1 at 1 minute and 2 at 5 minutes). Two units of fresh frozen plasma were transfused (due to her bleeding tendency associated with FVII deficiency) just prior to surgery. Intraoperatively, intrauterine balloon tamponade was performed because postpartum hemorrhage combined with uterine atony after placental delivery was observed. Total blood loss during surgery was estimated to be 900 mL.

**Figure 1.**
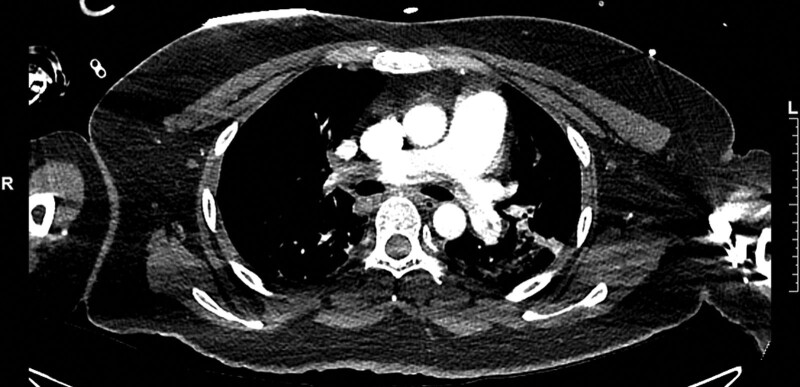
Axial computed tomography image showing thrombosis in the right main pulmonary artery.

**Figure 2.**
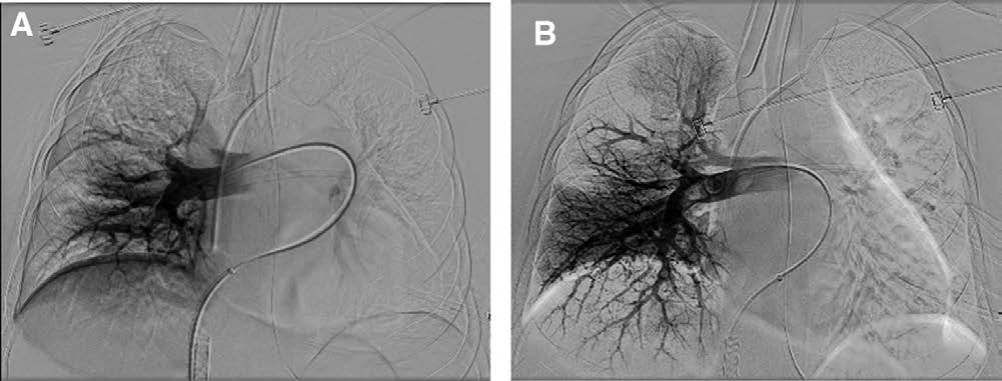
(A) Initial angiography of the right pulmonary artery demonstrating the presence of a thrombus within the right main pulmonary artery and the upper lobar branches. (B) Angiography after catheter-guided mechanical thrombectomy shows improvement in obstruction and perfusion of the right pulmonary artery and branches.

There was not much additional blood loss postoperatively, and the patient’s condition remained stable. The day after surgery, after the intrauterine balloon and Foley catheter were removed, the moment she arose from the bed, she experienced a sudden loss of consciousness and cardiac arrest. Cardiopulmonary resuscitation was performed once, and spontaneous circulation was restored. She was hypoxic on room air (PaO_2_, 75%) and tachycardic (146 beats/min), with metabolic acidosis (pH 7.29) and elevated lactate (3.0 μmol/L), suggestive of tissue hypoperfusion. The patient was intubated and moved to the intensive care unit, where she was administered vasopressors, and mechanical ventilation was initiated. A bedside echocardiogram demonstrated a dilated right ventricle with evidence of acute right ventricular pressure overload and a D-shaped left ventricle. The patient was then taken for enhanced chest computed tomography (CT), which showed massive pulmonary thromboembolism in the main, lobar, and segmental pulmonary arteries (Fig. 1). However, deep vein thrombosis was not clearly observed on Doppler ultrasound. Considering the risk for recurrent cardiac arrest because of her persistent low blood pressure (88/52 mm Hg) and tachycardia (121bpm), venoarterial ECMO was initiated. Heparinization was performed for anticoagulation via a loading dose and continuous venous infusion. With the support of ECMO (systemic heparin, flow rate 2.5 L/minutes), catheter-directed mechanical thrombectomy (Angiojet Zelante [8F], Boston Scientific, Marlborough, MA) was performed. Follow-up pulmonary angiography after the procedure revealed a significant decrease in thrombus volume (Fig. 2), and the patient’s circulation stabilized without vasopressor administration. After 2 days, RV dysfunction and the D shape of the left ventricle normalized on follow-up echocardiogram, so ECMO was discontinued. After 3 days, improvement of the PE was observed on follow-up enhanced chest CT, and focal residual deep venous thrombosis was identified in the left popliteal vein on enhanced CT venography. The patient was switched from IV heparin to oral rivaroxaban. She recovered well except for a gait disturbance caused by lumbosacral plexopathy. After strength and gait training, she was discharged in a stable condition 30 days postpartum. There were no major sequelae over 2 months of follow-up.

## 3. Discussion

This case demonstrated the risk of massive PE following premature rupture of the membranes and cesarean delivery in the context of FVII deficiency and high international normalized ratio. To the best of our knowledge, this is the first report of PE in a postpartum woman with FVII deficiency.

Women of reproductive age with inherited FVII deficiency have a high-risk of heavy menstrual bleeding, ovulation-related ovarian bleeding, and postpartum hemorrhage.^[10]^ However, because FVII deficiency is rare, large-scale studies on obstetric and gynecological outcomes in women with FVII deficiency are lacking, and standardized management strategies and specific guidelines have not been established.

Generally, there have been reports of elevated levels of FVII during pregnancy,^[11,12]^ as well as several reports of thrombosis in the context of FVII deficiency.^[3,4,13]^ A case of portal vein thrombosis discovered incidentally during pregnancy has also been reported.^[14]^ These observations indicate that FVII deficiency may not always offer protection against thrombotic events.

PE, most commonly originating from deep vein thrombosis in the legs, results in impaired pulmonary circulation and right-sided heart dysfunction. Previous case reports and reviews have reported a maternal survival rate of 85% in patients treated with, for example, thrombolysis, percutaneous or surgical thrombectomy, and ECMO.^[15]^

The incidence of pregnancy-related venous thromboembolism (VTE) is between 1/1000 and 1/2000 deliveries, which is 4 to 5 times higher than the incidence among nonpregnant women of the same age.^[16]^ The most important individual risk factors for pregnancy-related VTE are a personal history of thrombosis and thrombophilia, and related obstetric factors include cesarean delivery, preeclampsia, obesity, and multiple pregnancies.^[17]^ Recently, PPROM has also been mentioned as a risk factor.^[18,19]^ Given the morbidity and mortality associated with thrombotic disease, low-molecular-weight heparin is recommended for a large proportion of birthing women;^[20]^ however, the American College of Obstetricians and Gynecologists limits the indication to a history of thrombosis or potent thrombophilia.^[17]^

FVII deficiency is regarded as a hemorrhagic disorder. Contrary to popular belief, FVII deficiency does not offer thrombosis protection. Thrombosis usually occurs with risk factors, such as surgery, immobilization, and FVII agent replacement therapy.^[2]^ Girolami et al^[21]^ reported the occurrence of PE in 11 patients receiving VII concentrate, and Ramdasse et al^[22]^ reported a patient who developed PE after femoral neck surgery; this patient also received recombinant FVII before and after surgery. Moreover, there have been reports of spontaneous PE without risk factors.^[4]^ A case of chronic portal vein thrombosis incidentally discovered during pregnancy^[14]^ and a case of superficial venous thrombosis discovered during the postpartum period^[13]^ have also been reported in FVII-deficient women. However, there is no standard practice for antithrombotic prophylaxis for the treatment of thromboembolic events associated with rare bleeding disorders.

## 4. Conclusion

Physicians should be aware that FVII deficiency does not prevent thrombosis. Moreover, because of the increased risk of thrombosis associated with pregnancy, our case highlights that obstetricians should be mindful of potential thrombosis in the presence of additional risk factors, such as cesarean delivery and premature rupture of membranes. Furthermore, it is necessary to establish clearer criteria for medical thromboprophylaxis through risk stratification efforts for pregnancy-related VTE.

## Author contributions

**Conceptualization:** Donghoon Kang, Ji Eun Park.

**Data curation:** Hojeong Cha, Iyun Kwon.

**Investigation:** Sung Eun Park, Jong-Hwa Ahn, Ji Kwon Park.

**Writing – original draft:** Donghoon Kang, Ji Eun Park.

**Writing – review & editing:** Donghoon Kang, Ji Eun Park.
